# Management of symptoms of suspected adenomyosis uteri using herbal medicine modified Bojungikgi-tang: a case report with ultrasound monitoring

**DOI:** 10.3389/fmed.2025.1679449

**Published:** 2025-10-15

**Authors:** Hyunsuk Park, Hyein Jeong, Kyeong Han Kim, Seung Jeong Yang, Stella Roh

**Affiliations:** ^1^Department of Preventive Medicine, College of Korean Medicine, Kyung Hee University, Seoul, Republic of Korea; ^2^Department of Preventive Medicine, College of Korean Medicine, Woosuk University, Jeonju, Republic of Korea; ^3^Department of Korean Medicine Obstetrics and Gynecology, College of Korean Medicine, Dongshin University, Naju, Republic of Korea; ^4^Kirin Korean Medicine Clinic, Incheon, Republic of Korea

**Keywords:** adenomyosis, Hochu-ekki-to, Bu-Zhong-Yi-Qi-Tang, ultrasonography, Korean traditional medicine, case reports

## Abstract

**Introduction:**

Adenomyosis is a chronic gynecological disorder characterized by the infiltration of ectopic endometrial tissue into the myometrium, affecting approximately 20–35% of women of reproductive age. Although hormonal therapy and surgery are common treatments, their side effects and impact on fertility highlight the need for alternative approaches. Recently, integrative medicine, including traditional Korean medicine, has gained attention. However, well-documented long-term case studies using objective imaging are limited.

**Methods:**

A 40-year-old woman with severe adenomyosis symptoms unresponsive to conventional therapies received long-term herbal medicine based on modified Bojungikgi-tang, with regular ultrasound monitoring and clinical assessments over 12 months.

**Results:**

After treatment, menstrual pain decreased from a numeric rating scale score of 7–8 to 1–2, hemoglobin normalized, and ultrasonography revealed reduced adenomyosis and improved uterine structure. The patient’s quality of life improved markedly with no adverse effects.

**Conclusion:**

This case suggests that herbal medicine may be a safe and effective option for managing adenomyosis, supporting further research into integrative treatment strategies.

## 1 Introduction

Adenomyosis uteri is a chronic gynecological disease characterized by the infiltration of ectopic endometrial tissue into the uterine myometrium and is estimated to occur in 20–35% of women of childbearing age (1). Adenomyosis causes symptoms such as chronic pelvic pain, severe menstrual pain, and hypermenorrhea. In particular, hypermenorrhea is accompanied by anemia, fatigue, and depression, which seriously impair the physical and mental quality of life of patients (2).

The primary treatment strategies for adenomyosis include hormonal therapies (such as oral contraceptives, progestins, and gonadotropin-releasing hormone agonists) and non-hormonal agents (such as tranexamic acid). Endometrial ablation or hysterectomy may be considered when medical management fails. Hormonal therapies can cause side effects such as an increased risk of thrombosis and decreased bone mineral density, while surgical approaches such as hysterectomy result in loss of fertility in women of reproductive age. Therefore, the choice of treatment should be made cautiously, considering the patient’s reproductive plans, age, and severity of symptoms (3–6).

Recently, interest in integrative medicine approaches that utilize traditional Korean medicine (TKM), such as herbal medicine, acupuncture, and dietary therapy, as complementary and alternative to conventional medicine has increased (7). According to a systematic literature review of Chinese clinical studies on adenomyosis (8), herbal medicine shows similar effects to conventional medicine in terms of pain relief and reduction of menstrual flow, and its clinical usefulness has been reported, especially in patients with low drug tolerance. Yu et al. (9), Shim et al. (10), and Lee et al. (11) reported cases of adenomyosis treated using integrative Korean medicine, including herbal medicine. However, many previous studies had limitations in evaluating the effects of herbal medicine alone due to the use of complex interventions, including combination therapy with acupuncture, and long-term follow-up data using imaging indices were also lacking.

Herein, we present the case of a patient with dysmenorrhea and menorrhagia due to adenomyosis who received long-term herbal medicine treatment. By analyzing clinical symptoms (menstrual flow and pain intensity), blood tests (hemoglobin level), and ultrasound findings (uterine size and echotexture changes) before and after treatment, we explored the potential therapeutic effects of herbal medicine and the usefulness of ultrasound monitoring. Through this study, we aimed to suggest the possibility of an integrative medical approach utilizing TKM for the treatment of adenomyosis and establish basic data for the design of future randomized controlled trials.

## 2 Case presentation

This retrospective case study was approved by the Institutional Review Board of Woosuk University Korean Medicine Hospital (no.: WSOH IRB H2506-03). The patient was informed that the ultrasound photos and treatment history would be reported without personal information. We obtained consent from the patient for the academic publication of this case report.

### 2.1 Patient information

The patient was a 40-year-old woman who presented at K-Korean Medicine Clinic on April 2, 2023 (Last Menstrual Period (LMP) March 25, 2023) with various symptoms related to adenomyosis. In 2016, she was informed at a gynecology clinic that “ultrasound findings indicated adenomyosis and ovarian cysts,” and she had no history of pregnancy or childbirth (nulligravida, nullipara). At the time of her visit, she was taking Yaz® (drospirenone 3 mg/ethinyl estradiol 0.02 mg; Bayer AG, Germany) at a dose of one tablet daily.

On the day of her visit, ultrasonography revealed a globally enlarged uterus with asymmetric thickening of the myometrium. According to MUSA 2022, multiple direct signs were seen, including myometrial cysts, hyperechoic islands, and echogenic subendometrial lines and buds. Indirect signs included asymmetrical myometrial thickening, fan-shaped shadowing, and translesional vascularity on color Doppler. The presence of direct and indirect signs supported a diagnosis of adenomyosis per MUSA guidelines.

Her main symptoms included severe dizziness (hemoglobin, 6–8 g/dL) and dysmenorrhea (numeric rating scale [NRS] score, 7–8) along with pallor, epigastric tenderness, indigestion, fatigue, and limb weakness. She frequently experienced right lower abdominal pain during ovulation, bloating, and constipation before menstruation. Her menstrual period lasted approximately 6–7 days, with a marked increase in bleeding and pain starting 12 h after onset. During heavy bleeding, she used 10–15 large straight-type diapers (Clean Bebe®, Yuhan-Kimberly, Korea) daily, requiring changes approximately every hour owing to the excessive menstrual flow. Large clots (≥2–2.5 cm, 2–3 per urination) and 2–3 episodes of flooding per cycle were noted, and the PBAC score was over 400, indicating severe heavy menstrual bleeding. The pain began in the paraspinal muscles and radiated to the lower abdomen and back. She took Pain Angel Lady Soft Capsule® (ibuprofen 400 mg, pamabrom 25 mg; JW Pharmaceutical, Korea) every 4 h, totaling approximately 12 doses during each menstrual period. During menstruation, she was unable to eat solid food because of the pain and could tolerate only a liquid diet. Her usual body weight was 51–52 kg, which decreased to 48–49 kg during menstruation.

In December 2021, she took the hormonal agent Visanne® (dienogest 2 mg; Bayer AG, Germany) for 1 month but discontinued it because of its side effects. She started taking Yaz® in March 2022 at a dose of one tablet daily. After starting Yaz®, her menstrual volume decreased by approximately 50%; however, her cycle became shorter and more irregular, and she continued to experience NRS 7–8 pain for 12–36 h from the onset of menstruation, with persistent dizziness. Although her daily life was somewhat manageable outside of her menstrual period after starting Yaz®, she continued to experience significant limitations during menstruation.

### 2.2 Treatments

The patient was prescribed herbal medicine and instructed to take it 30 min after meals, 2–3 times daily. Modified Bojungikgi-tang (Bu-Zhong-Yi-Qi-Tang, BZYQT) was prescribed according to the patient’s symptoms. Asini Corii Colla, Rehmanniae Radix Recens, Zingiberis Rhizoma, Poria Sclerotium, Atractylodes Rhizome, and Cervi Cornus Colla were additionally included in the prescription based on the clinical judgment of the Korean medicine doctor. The ingredients and dosages of the prescribed herbal medicines are listed in the table below (Table 1). To maximize the therapeutic effect and prevent side effects, the patient was instructed to avoid consuming caffeine (coffee, black tea, etc.), dairy products, and flour-based foods as much as possible while taking the herbal medicine.

**Table 1:** Composition and quantities of herbal medicine.

Medicinal name	Chinese name	Scientific name	Dosage (g/Po)
Astragali Radix	黃芪	*Astragalus membranaceus*	4
Angelicae Gigantis Radix	當歸	*Angelica gigas*	4
Cervi Cornus Colla	鹿角膠	*Cervus elaphus*	1.67
Ginseng Radix	人蔘	*Panax ginseng*	1.33
Atractylodes Rhizome	白朮	*Atractylodes macrocephala*	1.33
Glycyrrhizae Radix et Rhizoma	甘草	*Glycyrrhiza uralens*	1.33
Asini Corii Colla	阿膠	*Equus africanus asinus*	1.33
Rehmanniae Radix Recens	生地黃	*Rehmania glutinosa*	1.33
Cimicifugae Rhizoma	升麻	*Cimicifuga heracleifolia*	0.67
Bupleuri Radix	柴胡	*Bupleurum falcatum*	0.67
Citri Unshii Pericarpium	陳皮	*Citrus unshiu*	0.67
Poria Sclerotium	茯苓	*Poria cocos*	0.67
Alismatis Rhizoma	澤瀉	*Alisma orientale*	0.67
Zingiberis Rhizoma	乾薑	*Zingiber officinale*	0.27

### 2.3 Ultrasound monitoring

For diagnostic evaluation, transabdominal ultrasonography was performed by a Korean Medicine doctor certified as a registered diagnostic medical sonographer in Obstetrics & Gynecology by the American Registry for Diagnostic Medical Sonography, with over 20 years of experience in ultrasonography. The ultrasound equipment used was a GE Healthcare LOGIQ P9, and the examination was performed using a C1-5 convex probe (4 MHz frequency). The probe was placed in the lower abdomen above the pubic bone and the uterus was examined longitudinally to obtain the optimal image. In cases of adenomyosis discovered during examination, the size was evaluated by measuring the maximum diameter in the horizontal and vertical directions.

### 2.4 Outcome measures

#### 2.4.1 Numeric rating scale (NRS)

The NRS is commonly used in healthcare settings for pain assessment, along with the visual analog scale. The NRS, a 0–10 pain scale in which higher scores indicate greater pain, was used at each outpatient visit.

#### 2.4.2 European quality of life-five dimension (EQ-5D-5L)

The EQ-5D-5L, a five-domain health-related quality of life measure, was used in this study, with higher scores indicating better quality of life. The index was calculated using the National Evidence-based Healthcare Collaborating Agency quality weighting formula, and assessments were conducted at the first visit, at the end of treatment, and 1 year after the end of treatment.

#### 2.4.3 Morphological uterus sonographic assessment 2022 (MUSA 2022)

The 2022 MUSA criteria classify ultrasound features of adenomyosis into direct signs (e.g., myometrial cysts, hyperechogenic islands, and echogenic subendometrial lines) and indirect signs (e.g., asymmetrical myometrial thickening, fan-shaped shadowing, irregular junctional zone).

#### 2.4.4 Pictorial blood loss assessment chart (PBAC) score

The Pictorial Blood Loss Assessment Chart (PBAC) is a scoring system developed as a semi quantitative evaluation of menstrual blood loss, which considers the number of sanitary products used, the degree to which these products are soiled with blood, the number and size of blood clots passed, and the number of flooding episodes.

### 2.5 Follow-up and outcome

The patient had severe menstrual pain, anemia, indigestion, and dizziness at the time of her first visit (April 2, 2023 [LMP; March 25, 2023]), making it difficult for her to carry out daily life activities to the point where she had to lie down for approximately 2 weeks out of the month. Her menstrual cycle was short (24–25 days) and an ultrasound examination showed that most of the posterior wall of the uterus was filled with adenomyotic tissue and the uterine anteroposterior (AP) length was 8.7 cm. Her EQ-5D-5L index was 0.288 points and her NRS score was 7–8.

On April 24, 2023, she started taking herbal medicine (modified BZYQT) three times daily after meals and reduced the dose of the oral contraceptive Yaz® she was taking to half a tablet daily. She reported slight improvements in physical strength, dizziness, and indigestion from the third day of taking the herbal medicine.

On June 11, 2023, after 6 weeks of taking the herbal medicine, the patient’s menstrual pain decreased to NRS 4–5, and the dosage of painkillers was reduced to 1–2 daily for only 2 days when the pain was severe. The intensity and frequency of back pain also decreased, and small meals and sleep were possible even on days when the menstrual pain was severe. The uterine AP length was 7.5 cm.

During gynecological examination in late August 2023, the patient’s hemoglobin level improved to 10.4, and ultrasound findings indicated regeneration of normal myometrial tissue, starting from the margins of the adenomyotic lesion.

On September 10, 2023 (LMP; September 6, 2023), herbal medicine intake was adjusted to twice daily.

On October 15, 2023 (LMP; October 7, 2023), the menstrual cycle gradually became longer, and ultrasound findings showed that the boundary between the adenomyotic lesion and the endometrium had become clearer, with an expansion of normal endometrial tissue and a reduction in the size of the ovarian cysts.

In November 2023, dizziness disappeared, her digestive function improved, and the amount of food consumed increased.

On December 3, 2023 (LMP; November 21, 2023), an ultrasound examination revealed a decrease in the extent of adenomyotic lesions and an increase in the extent of normal tissue. The uterine AP length was 7.62 cm. In addition, during a health checkup conducted in the same month, the hemoglobin level normalized to 11, and during a gynecological examination in February 2024, both the size of the ovarian cysts and the extent of adenomyosis had decreased.

By May 26, 2024 (LMP; May 10, 2024), the menstrual cycle had normalized to 28–29 days, the menstrual period lasted 4–5 days, and the menstrual flow decreased to a point where only 5–6 large sanitary pads were used for 2–3 days of heavy menstrual flow. No clots or flooding were observed, and the PBAC score was approximately 55, which is within the normal range. Menstrual pain was greatly reduced to NRS 1–2, and the number of painkillers used was reduced to 1–2. The degree and duration of back pain also significantly reduced, and normal eating and activities of daily living were possible even during menstruation. Secondary symptoms such as dizziness, decreased physical strength, and indigestion also significantly improved, and normal activities of daily living were possible, unlike before when she had to lie down for more than half a month. In addition, ultrasound according to MUSA 2022 showed myometrial cysts and hyperechoic islands present but reduced in size and number; subendometrial echogenic lines and buds were not detected, with a distinct endometrium separate from the myometrium. The uterine anterior wall ratio was increased compared to before treatment, and atypical intramyometrial shadows decreased. Color Doppler revealed blood flow crossing the lesion at some distance from the junctional zone. The uterus remained asymmetrically enlarged but the fundus partially regained normal shape. The endometrium–myometrium junction was relatively regular with normal tissue pattern and continuous echoes, and the junctional zone thickness averaged 15 mm. The mean EQ-5D-5L index was 0.877 points, and she stopped taking herbal medicine thereafter. Changes in ultrasound findings based on the MUSA 2022 criteria are summarized in Supplementary Tables 1, 2. After discontinuation of herbal medication, the liver function test (AST 18 IU/L, ALT 6 IU/L, γ-GTP 6 IU/L) and renal function test (creatinine 0.6 mg/dL, eGFR 113 mL/min/1.73m²) were all within normal ranges.

On March 9, 2025 (LMP; February 23, 2025), the patient went on with her normal daily life activities with negligible menstrual pain; therefore, she did not require painkillers but remained on Yaz® oral contraceptive (half tablet). The uterine AP length was 7.28 cm. The EQ-5D-5L index was maintained at 0.877 points.

No side effects or unexpected adverse reactions were observed during the treatment. The patient had a high level of compliance with the herbal medicine treatment, and the treatment effect was continuously monitored through consistent medication for more than 12 months and regular visits.

**Figure 1:**
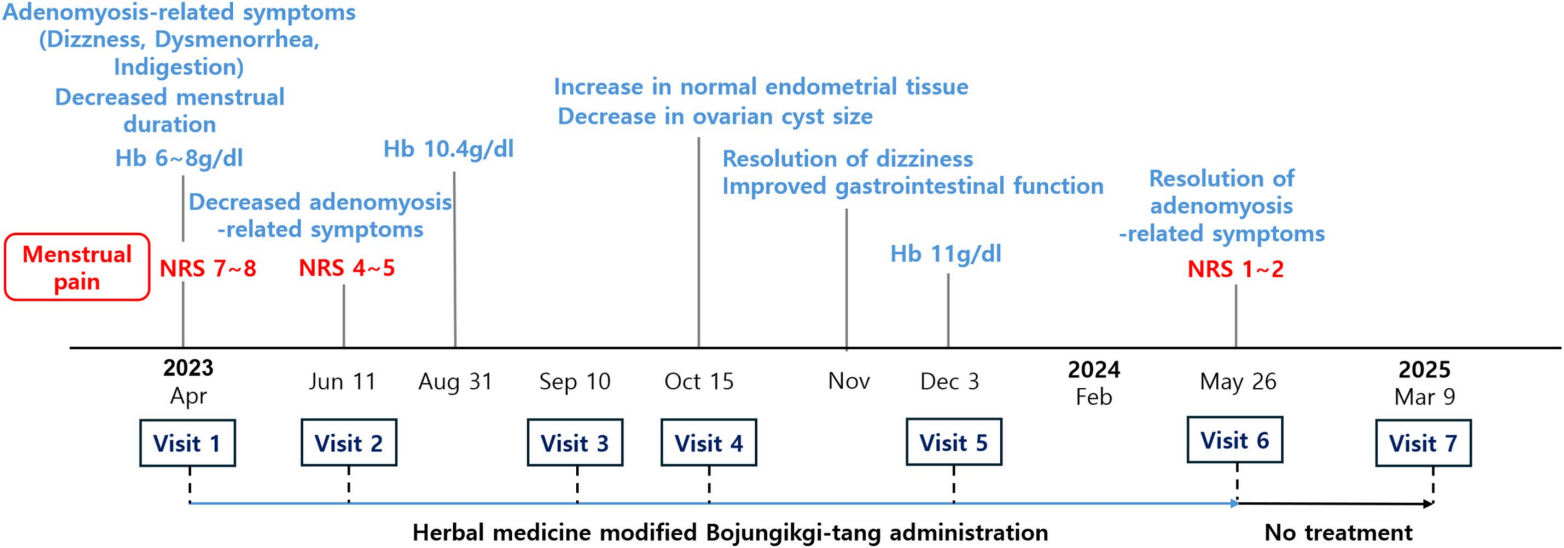
Timeline of major clinical events and treatment course.

**Figure 2:**
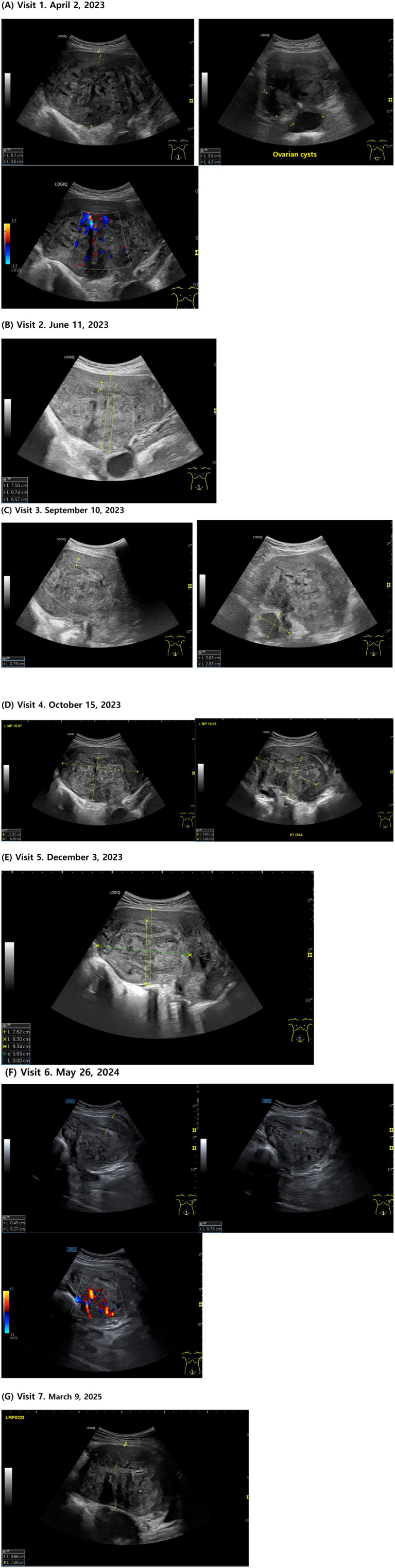
Transabdominal ultrasound images showing adenomyosis. **(A)** Visit 1. April 2, 2023. **(B)** Visit 2. June 11, 2023. **(C)** Visit 3. September 10, 2023. **(D)** Visit 4. October 15, 2023. **(E)** Visit 5. December 3, 2023. **(F)** Visit 6. May 26, 2024. **(G)** Visit 7. March 9, 2025.

The visit and treatment schedule of this patient are shown in Figures 1, 2 and Table 2.

**Table 2:** Detailed clinical measurements and observations at each visit.

Visit	Visit date	Menstrual pain (NRS)	PBAC Score	Changes in main symptoms
1	April 02, 2023	7 ~ 8	> 400	Herbal medicine started (TID), Apr 2023 The mean EQ-5D-5L index was 0.288 points
2	June 11, 2023	4 ~ 5		Decrease in dysmenorrhea NRS score to 4–5 Reduced analgesic intake Able to eat and sleep even on days with severe dysmenorrhea
3	September 10, 2023			Dose of herbal medicine adjusted to twice daily
4	October 15, 2023	2 ~ 3		Gradual lengthening of menstrual cycle Clearer delineation between adenomyotic lesions and endometrium Decreased size of ovarian cysts
5	December 3, 2023			Reduced extent of adenomyosis; increased normal myometrial tissue Hemoglobin level improved to 11 g/dL
6	May 26, 2024	1 ~ 2	55	Menstrual duration reduced by 4–5 days; cycle regularized to 28–29 days Significant improvement in associated symptoms: dizziness, fatigue, and indigestion Normal dietary intake and daily activities maintained during menstruation
7	March 9, 2025			The patient continued her normal daily life Menstrual pain had almost disappeared The mean EQ-5D-5L index was 0.877 points

### 2.6 Patient perspective

After completing the treatment, the patient said, “As an unmarried woman, I am very satisfied because all my symptoms improved with herbal medicine without surgery, and my quality of life has improved, including my ability to work.”

## 3 Discussion

This case report describes a patient who suffered from adenomyosis-related symptoms for 5 years and did not respond to conventional treatments. The patient was treated with modified BZYQT, and her progress was monitored using ultrasonography. We comprehensively analyzed the menstrual volume, pain intensity, blood test results, and ultrasound images before and after treatment to explore the clinical utility of herbal medicine and the validity of radiological monitoring.

BZYQT comprises Astragali Radix, Ginseng Radix, Angelicae Gigantis Radix, Atractylodis Rhizoma Alba, and other herbs (12). Systematic reviews and clinical studies have reported that BZYQT improves hemoglobin and hematocrit levels in patients with chronic fatigue syndrome and anemia (13), and it is recommended in various clinical studies and guidelines for improving conditions such as immune dysfunction, chronic indigestion, and general fatigue (14, 15). In addition to these general applications, BZYQT has been widely used in gynecological and obstetric conditions. According to Ni et al. (16), BZYQT has been reported effective in polycystic ovary syndrome (PCOS) and other hormone-related gynecological disorders. Based on this evidence, BZYQT was selected as the primary prescription for this patient.

Astragali Radix, the main herb in this prescription, is traditionally used to promote tissue regeneration, wound healing, and mucosal repair (17). Recent pharmacological studies have demonstrated its physiological effects on angiogenesis, fibroblast proliferation, and collagen synthesis, mediated through modulation of key signaling pathways such as phosphatidylinositol 3-kinase (PI3K)-protein kinase B (Akt), mitogen-activated protein kinase (MAPK), and forkhead box O (FoxO) (18, 19). Notably, another pharmacological research has also reported that Astragali Radix can induce ovarian *β*-oxidation and suppress estrogen-dependent endometrial proliferation, thereby improving estrogen-related gynecological pathologies (20). This suggests that Astragalus may influence estrogen-responsive tissues through both metabolic and receptor-mediated regulation, supporting its therapeutic relevance in conditions such as adenomyosis. Rehmanniae Radix Recens has the dual action of replenishing blood and generating body fluids and has traditionally been used to treat hemorrhagic diseases such as metrorrhagia. It contributes to anti-inflammatory and metabolic regulation by promoting apoptosis in damaged tissues and supporting cellular repair, and has effects on various inflammatory and metabolic diseases (21).

Asini Corii Colla and Cervi Cornus Colla are animal-derived gelatin products extracted from donkey hide and deer antler, respectively, and are rich in collagen protein, which promotes tissue regeneration, hematopoiesis, hemostasis, and blood supplementation (22). Asini Corii Colla has been widely used for hemorrhagic diseases, anemia, debility, and wound healing and promotes erythropoiesis and improves hemoglobin levels (23, 24). Cervi Cornus Colla is also highly effective for hemostasis and blood supplementation and has been used to treat chronic bleeding and debility in female reproductive diseases (25).

In this case report, Rehmanniae Radix Recens, Asini Corii Colla and Cervi Cornus Colla were added to BZYQT to further enhance the effects of the original formula. By adding medicinal herbs with immune-regulating, anti-inflammatory, and blood-replenishing effects, modified BZYQT is thought to have helped alleviate the inflammatory environment in the uterus while promoting blood circulation and helping tissue recovery in the lesion area.

The decrease in the extent of myometrial lesions observed in the present study suggests that herbal medicine suppresses abnormal endometrial proliferation into the myometrium. Moreover, the normalization of menstrual flow and ovulation and improvement in menstrual pain demonstrate that herbal medicine treatment is effective not only in controlling myometrial lesions but also in improving physiological functions throughout the menstrual cycle. Further high-quality evidence-based studies are required to confirm the efficacy.

Herbal medicine has long been used for estrogen-dependent disorders such as adenomyosis and endometriosis. These conditions frequently coexist and share key pathophysiological processes, including inflammation, immune dysregulation, tissue invasion, and hormone dependence. Recent systematic reviews and clinical trials have provided increasing evidence of its efficacy and safety, suggesting it as a complementary and alternative option for estrogen-dependent disorders (8, 26, 27). Although its efficacy is believed to be mediated by modulation of estrogen receptor activity and anti-inflammatory effects (28), clear evidence for direct reductions in estrogen levels remains lacking. Further large-scale clinical trials and pharmacological studies are needed to confirm the mechanisms and efficacy of herbal medicine.

No side effects or adverse events were reported during the treatment period. and the patient was continuously monitored using ultrasound to closely check the safety and treatment progress. Several clinical studies have also reported that BZYQT has been used safely without serious adverse events (13–15). These findings are expected to objectively support the safety of Korean medicine interventions and help increase the clinical reliability and legitimacy of treatments (29).

Adenomyosis is increasingly recognized in adolescents and young girls, where early onset symptoms can lead to significant quality-of-life impairment and future fertility concerns (1, 30). Herbal medicine may be considered a promising complementary or alternative treatment method in this population, offering long-term symptom control, reducing reliance on hormonal therapy, preserving fertility, and avoiding hysterectomy.

This study presents a single case report, therefore a possibility of confounding factors due to lifestyle changes (reduction in caffeine and processed food intake), continuous progestin-based oral contraceptive use, and the patient’s age approaching menopause; all of which could have influenced the clinical outcome. Hence, this study had limitations in clarifying the sole effect of modified BZYQT. Also, hormone test data and blood test results that could objectively assess the patient’s endocrine status and treatment response should be included in future studies. Nevertheless, this case report demonstrates the possibility of significant clinical improvement without hysterectomy while preserving fertility in patients with adenomyosis. Although the results of this study are encouraging, additional case series and large-scale randomized controlled trials should be conducted to verify the therapeutic effects of herbal medicines on estrogen-dependent diseases, including adenomyosis.

## 4 Conclusion

In this case report, modified BZYQT was prescribed to a patient with adenomyosis, and it effectively improved adenomyosis and its related symptoms. Following herbal medicine treatment, ultrasonography revealed decreased abnormal tissue in the myometrium, and all symptoms caused by adenomyosis were alleviated. In addition, the treatment effect was continuously maintained during the one-year follow-up observation period after the end of treatment.

## Data Availability

The original contributions presented in the study are included in the article/Supplementary material, further inquiries can be directed to the corresponding author.
