# Supplementary material for: Management of symptoms of suspected adenomyosis uteri using herbal medicine modified Bojungikgi-tang: a case report with ultrasound monitoring
Source: Front Med (Lausanne). 2025 Oct 15;12:1679449. doi: 10.3389/fmed.2025.1679449 (PMC12568549; doi:10.3389/fmed.2025.1679449)
Supplement: Supplementary file 1 [file Data_Sheet_1.PDF]

Supplementary Table 1. Ultrasound findings on 2 April 2023 evaluated by MUSA 2022 criteria

	**Ultrasound findings on 2 April 2023**
**Doppler image**	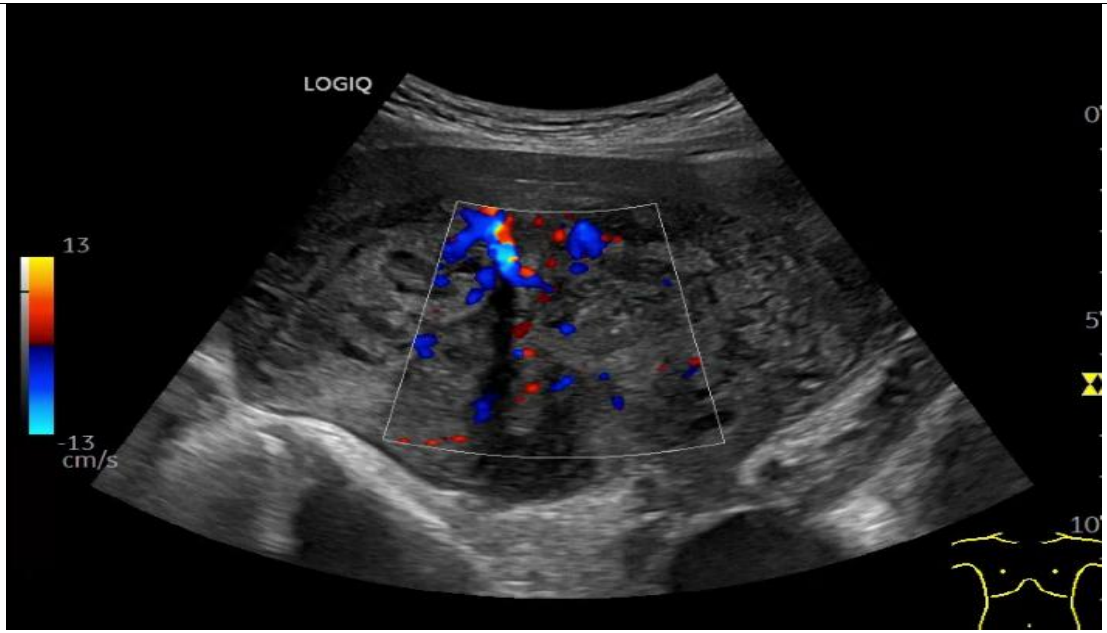
**Direct signs**	· Myometrial cysts: Multiple cysts present, with relatively increased size compared to post-treatment.<br>· Hyperechoic islands: Multiple islands present.<br>· Subendometrial echogenic lines and buds: Detected.
**Indirect signs**	· Asymmetrical thickening of the myometrium: The proportion of the uterine posterior wall is significantly greater than that of the anterior wall.<br>· Fan-shaped shadowing: Numerous atypical intramyometrial shadows are observed.<br>· Translesional vascularity: On color Doppler, blood flow traversing the lesion originates from the myometrial junction.<br>· Globular uterus: The overall uterine shape is globular or asymmetrically enlarged.<br>· Irregular/interrupted junctional zone: The junctional zone appears irregular or interrupted with an average thickness of 5 mm.

Supplementary Table 2. Ultrasound findings on 26 May 2024 evaluated by MUSA 2022 criteria

	**Ultrasound findings on 26 May 2024**
**Doppler image**	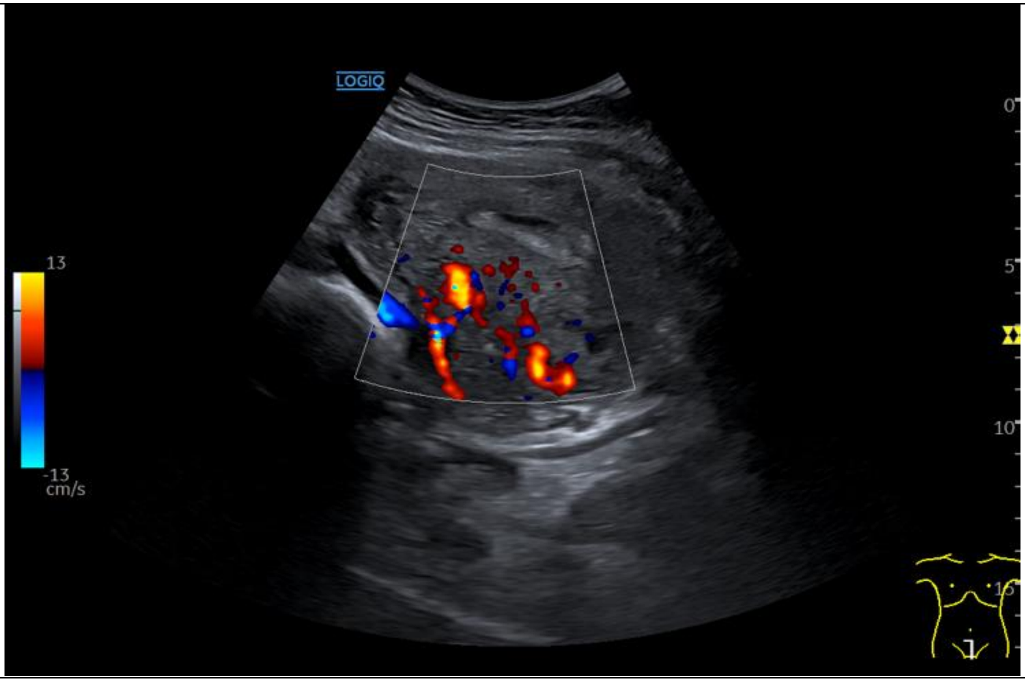
**Direct signs**	• Myometrial cysts: Present but reduced in number and size compared to before treatment.<br>• Hyperechoic islands: Present but the area of presence is reduced compared to before treatment.<br>• Subendometrial echogenic lines and buds: Not detected; a distinct endometrium separate from the myometrium is observed.
**Indirect signs**	• Asymmetrical thickening of the myometrium: The uterine wall remains asymmetrically thickened, but the ratio of the anterior wall is increased compared to before treatment.<br>• Fan-shaped shadowing: Atypical intramyometrial shadows remain but decreased compared to before treatment.<br>• Translesional vascularity: Blood flow traversing the lesion is observed on color Doppler but occurs at some distance from the myometrial junction.<br>• Globular uterus: The uterus remains asymmetrically enlarged, but the fundus region partially regains a normal shape.<br>• Irregular/interrupted junctional zone: The junction between the endometrium and myometrium is relatively regular with normal tissue pattern and continuous regular echoes (no interruption) with an average junctional zone thickness of 15 mm.
